# The risk of sustained sexual transmission of Zika is underestimated

**DOI:** 10.1371/journal.ppat.1006633

**Published:** 2017-09-21

**Authors:** Antoine Allard, Benjamin M. Althouse, Laurent Hébert-Dufresne, Samuel V. Scarpino

**Affiliations:** 1 Centre de Recerca Matemàtica, Edifici C, Campus Bellaterra, Bellaterra, Barcelona, Spain; 2 Institute for Disease Modeling, Bellevue, Washington, United States of America; 3 University of Washington, Seattle, Washington, United States of America; 4 New Mexico State University, Las Cruces, New Mexico, United States of America; 5 Santa Fe Institute, Santa Fe, New Mexico, United States of America; 6 University of Vermont, Burlington, Vermont, United States of America; 7 Northeastern University, Boston, Massasschusetts, United States of America; University of California, Los Angeles, UNITED STATES

## Abstract

Pathogens often follow more than one transmission route during outbreaks—from needle sharing plus sexual transmission of HIV to small droplet aerosol plus fomite transmission of influenza. Thus, controlling an infectious disease outbreak often requires characterizing the risk associated with multiple mechanisms of transmission. For example, during the Ebola virus outbreak in West Africa, weighing the relative importance of funeral versus health care worker transmission was essential to stopping disease spread. As a result, strategic policy decisions regarding interventions must rely on accurately characterizing risks associated with multiple transmission routes. The ongoing Zika virus (ZIKV) outbreak challenges our conventional methodologies for translating case-counts into route-specific transmission risk. Critically, most approaches will fail to accurately estimate the risk of sustained sexual transmission of a pathogen that is primarily vectored by a mosquito—such as the risk of sustained sexual transmission of ZIKV. By computationally investigating a novel mathematical approach for multi-route pathogens, our results suggest that previous epidemic threshold estimates could under-estimate the risk of sustained sexual transmission by at least an order of magnitude. This result, coupled with emerging clinical, epidemiological, and experimental evidence for an increased risk of sexual transmission, would strongly support recent calls to classify ZIKV as a sexually transmitted infection.

## Author summary

The national and international community is grappling with how to respond to the ongoing Zika virus outbreak. One of the most uncertain aspects of this disease is its potential for sustained sexual transmission. Recent studies have suggested that there are large differences in the age- and sex-specific Zika virus attack rates—with women of childbearing age having the highest incidence of infection—and that the risk of sustained sexual transmission may be low. Here we investigate the novel epidemiological behavior of an infection that is transmitted both sexually and by a mosquito vector. Using data-driven simulations, we demonstrate how conventional methods can substantially underestimate the risk of sustained sexual transmission for Zika outbreaks. More specifically, we find that the threshold for large-scale, sustained sexual transmission could easily be underestimated by a factor of ten or higher using existing models. Finally, we discuss how such an underestimate will lead to ineffective responses and a drastic underestimation of the risk associated with Zika persistence and re-emergence.

## Introduction

Recent epidemiological [1, 2, 3, 4, 5], clinical [6, 7, 8], and experimental [9, 10, 11] evidence suggests that Zika virus (ZIKV) poses a risk for sexual transmission. These findings, which include anecdotal examples of sexual transmission in humans [1], substantial asymmetries in observed age- and sex-specific ZIKV attack rates across Central and South America [3], persistent viral shedding in bodily fluids [6] leading to transmission months after initial symptom onset [12], and high rates of sexual transmission in mouse [9] and macaque [10] animal models, have led to an ongoing debate about whether to classify ZIKV as a sexually transmitted infection (STI) [13, 14, 15, 16, 17] and recommendations from the US CDC for couples to take precautions after returning from ZIKV endemic areas [18]. From a public health perspective, it is critically important to determine whether sexual transmission of ZIKV is likely to be sporadic or sustained. Sustained sexual transmission would have the immediate effect of increasing the number of cases during local outbreaks, and a longer-term effect if it led to endemic maintenance of ZIKV in regions that would otherwise experience only sporadic outbreaks. Lastly, emerging evidence from experimental transmission studies in mice suggest that sexual transmission may lead to higher rates of ZIKV-associated birth defects such as microcephaly [19]. Mathematical transmission models allow us to formally evaluate and quantify the risk of sustained sexual transmission of ZIKV. Here, we use data-driven simulations of such models to inform estimates of the risk of sustained sexual transmission of ZIKV.

An infectious disease is unlikely to generate a large outbreak if its basic reproductive number, *R*_0_, is less than one. With *R*_0_ ≥ 1, each infected individual will on average give rise to at least one additional case and the outbreak will be self-sustaining in a large population; while the transmission process dies out if *R*_0_ < 1 as individuals give rise to less than one additional case on average. Therefore, public health officials are interested in both estimating *R*_0_ and evaluating which interventions are most likely to reduce it. In the unique situation of sexual transmission of ZIKV, we must consider the following three issues when calculating *R*_0_ and constructing mathematical/computational transmission models: (1) individuals infected by the mosquito vector are not necessarily part of the branching process over the network of sexual contacts; (2) all infections are not equivalent—there is a strong asymmetry in sexual transmission as men are infectious for much longer than women [8]; and (3) symptoms, and thus the probability of detection, are also heterogeneous [20].

To date, the majority of models published on ZIKV spread have focused exclusively on mosquito-driven transmission and can be roughly divided into statistical [21, 22, 23, 24, 25], differential equation-based [26, 27, 28, 29], and agent-based models [30]. The studies that included sexual transmission have all made a number of simplifying assumptions, which we relax in our investigation. For example, Gao et al. (2016) assumed that transmission occurred via monogamous, heterosexual partnerships with equal risk of transmission from either males or females [31]. As we detail below, these assumptions are both incompatible with existing data on ZIKV sexual transmission [7, 8] and, more importantly, will likely underestimate the risk of sustained sexual transmission [32, 33]. Additionally, Brauer et al. (2016) and Agusto et al. (2017) found that including sexual transmission allows for an endemic equilibrium with *R*_0_ < 1 for sexual transmission [34, 35]; however, both assumed that sexual contacts occur randomly and are equal in number across individuals. Again, these assumptions are both untrue of sexual contact networks [36] and will likely underestimate the risk of transmission [32, 33].

Aside from complications due to the vector and modeling assumptions, the risk of sexual transmission of ZIKV is itself confounded due to vast differences in the infectivity and longevity of ZIKV in semen [8] vs. vaginal fluids [7]. The epidemiological consequences of this asymmetry partially explains why females are significantly less likely to transmit ZIKV to their sexual partner than males [7, 8] and evidence exists for male-to-male sexual transmission [4], despite no active screening in communities of men-who-have-sex-with-men (MSM). This asymmetry in transmission has also been demonstrated in a mouse model of sexual transmission, where males were infectious for significantly longer than females and >70% of sexual contacts between infected males and uninfected females led to transmission [9]. Additionally, in a mouse exposure trial, 100% of intrarectal exposures led to infection, as compared to 50% of intravaginal exposures [10]. The goal of this work is therefore to relax the aforementioned modeling assumptions and include asymmetry in infectivity/viral longevity between males and females in order to study how the unique nature of ZIKV transmission, i.e. mosquito- and sexually-driven, interacts with heterogeneity in sexual contact networks and transmission asymmetry to determine both the expected epidemic size and probability of endemic establishment.

## Methods

To investigate the effect of relaxing the aforementioned assumptions, we employ the modeling approach of asymmetric bond percolation on random sexual networks [32, 37]. At its core, this approach uses synthetic random sexual networks generated as follows. (1) A population of *N* individuals is assumed to be equally split between men and women, of which 5% are homosexuals, 3% are bisexuals and 92% are heterosexuals (conservative values determined from published estimates [38, 39, 40]), thus yielding six types of nodes (individuals). (2) To account for heterogeneity in the number of contacts, individuals are assigned a number of sexual contacts drawn from distributions conservatively extrapolated from Ref. [39] (for homosexual contacts) and Ref. [38] (for heterosexual contacts). (3) Sexual partnerships are then formed at random following the individuals’ sexual orientation. For instance, bisexual women choose their partners randomly in the pools of heterosexual men, bisexual men and women, and homosexual women. (4) Finally, each sexual partnership is translated into a set of directed links between the two partners depending on whether transmission would eventually occur should one of them become infected by ZIKV. More precisely, in a sexual partnership between nodes *i* and *j*, a directed link from *i* to *j* is added with probability *T* if *i* is a man, or with probability *T*/*ε* if *i* is a woman to account for the observed strong asymmetry in the probability of transmission (i.e., women are *ε* times less likely to transmit ZIKV than men). Since both directions are considered, any partnership (*i*, *j*) can therefore lead to (i) no link, (ii) a directed link from *i* to *j*, (iii) a directed link from *j* to *i* or (iv) two directed links running in opposite directions. The resulting semi-directed network then corresponds to the sexual transmission paths that ZIKV will follow should a node get infected by a mosquito. The conclusions of this paper are based on data from numerical simulations and from direct measurements of these synthetic sexual networks. Note that these data were averaged over many realizations of the networks.

Conceptually, we decompose the traditional measure of *R*_0_ for a sexually transmitted infection into three distinct measures of secondary case potential on which we will elaborate in the Results section:

- $R_{0}^{\text{total}}$: Conceptually similar to the traditional measure of *R*_0_ or roughly, the average number of secondary sexual infections directly caused by any infected individual. While this measure includes secondary sexual infections from both vector- and non-vector-derived cases, it excludes secondary infections caused by ongoing vector-mediated transmission;
- $R_{0}^{\text{sexual}}$: Average number of secondary infections directly caused by an individual infected sexually, i.e. individuals infected by the vector are excluded;
- $R_{0}^{\text{MSM}}$: Similar to $R_{0}^{\text{sexual}}$ but only taking into account individuals with ongoing sexual contacts in the men-who-have-sex-with-men (MSM) community.

Additionally, we characterize the overall threat of ZIKV as an STI by measuring the potential prevalence of the epidemic and potential for sustained, endemic transmission. This quantity corresponds to the macroscopic fraction of nodes that will eventually be reached by following any link from a given node (assuming that patient zero was infected by a mosquito). In practice, this fraction is considered macroscopic if it is greater than *N*/500.

This modeling approach uses asymmetric bond percolation on static networks as a “first order” approximation of a spreading dynamic in which true sexual contacts change over time and where timescales, both of the contact-swapping dynamics and the infectious period, greatly influence the outcomes. Although seemingly simplistic, this approach nevertheless captures key features of ZIKV outbreaks, namely asymmetric probability of transmission and heterogeneous number of sexual contacts. An important extension of our work would be to study ZIKV transmission on a dynamic contact network; however, the currently available data on sexual contact networks is likely too coarse-grained for such an analysis to be empirically grounded. It is also worth mentioning that the modeling approach used in this study typically applies when there is only a small number of initial seeds (i.e., mosquito transmission events). While this makes our conclusions more applicable in regions without a viable mosquito vector but where people may bring back ZIKV with them after a trip to endemic regions, recent results showing an important asymmetry in incidence between men and women [3] strongly suggest that our conclusions are relevant for endemic regions as well.

## Results

### An adequate threat assessment tool

While it is tempting to evaluate the sexual *R*_0_ $\left(R_{0}^{\text{sexual}}\right)$ of ZIKV by averaging the number of sexual transmissions caused by all known infected individuals—which was the approach taken by Brauer et al. (2016) and Agusto et al. (2017)—it is not correct when transmission occurs on real-world sexual contact networks, which are notoriously heterogeneous [36]. Therefore, to estimate $R_{0}^{\text{sexual}}$ correctly, we need to evaluate the number of secondary infections caused by individuals who were infected sexually. The intuition behind this observation is that individuals infected by a mosquito will be “randomly” drawn from the total population (and thus have a number of sexual partners drawn from a distribution {*p*_*k*_}), whereas individuals infected sexually must be conditioned on having at least one contact. As a result of this conditioning, the sexually infected person is already 10 times more likely to have 10 contacts than only one contact (their number of sexual partners is thus drawn from a distribution proportional to {*kp*_*k*_}). This distinction between mosquito- and sexually-infected individuals derives from the classic network theory phenomenon of “your friends have more friends than you do” [41]. Note that this distinction does not imply that sexually-infected individuals have more heterogeneity in social connections than mosquito-infected individuals, but rather means that the dynamics of sexual infections are biased towards more connected individuals, which then implies that individuals infected sexually have, on average, a higher opportunity for subsequent secondary transmission.

**Fig 1:**
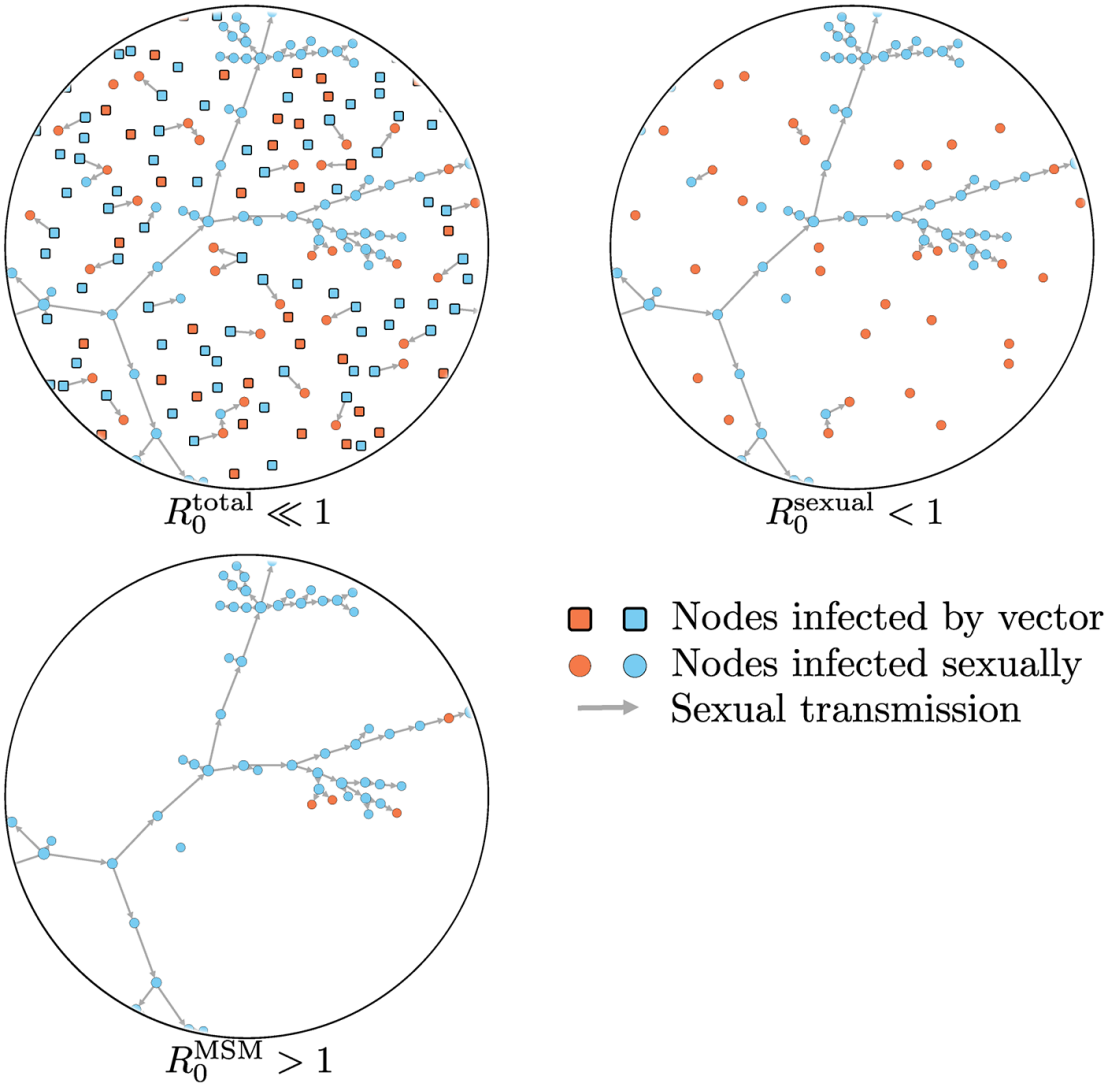
Women and men are shown in orange and blue, respectively, with squares indicating vector-infected individuals and circles corresponding to infections acquired sexually. Since most vector-infected individuals typically transmit ZIKV to 0 or 1 sexual partners, a biased estimate of the relevant reproductive number for sexual transmission risk ($R_{0}^{\text{total}}$), will be obtained when considering all infections, thus inaccurately suggesting that ZIKV cannot be a self-sustaining STI (one would correctly determine that $R_{0}^{\text{total}}⪡1$; however, this quantity underestimates the risk of sexual transmission). Considering only transmissions caused by sexually-infected individuals corrects for the bias, but still may not provide a complete assessment of the threat posed by ZIKV as an STI ($R_{0}^{\text{sexual}}<1$). In fact, because of the highly asymmetric sex-dependent probability of transmission of ZIKV, an epidemic is likely to occur in the men-who-have-sex-with-men (MSM) community with some spillover transmission to the population not active in the MSM community, a situation that can only be modeled through a well-suited community-specific reproductive number (e.g., $R_{0}^{\text{MSM}}>1$).

**Fig 2:**
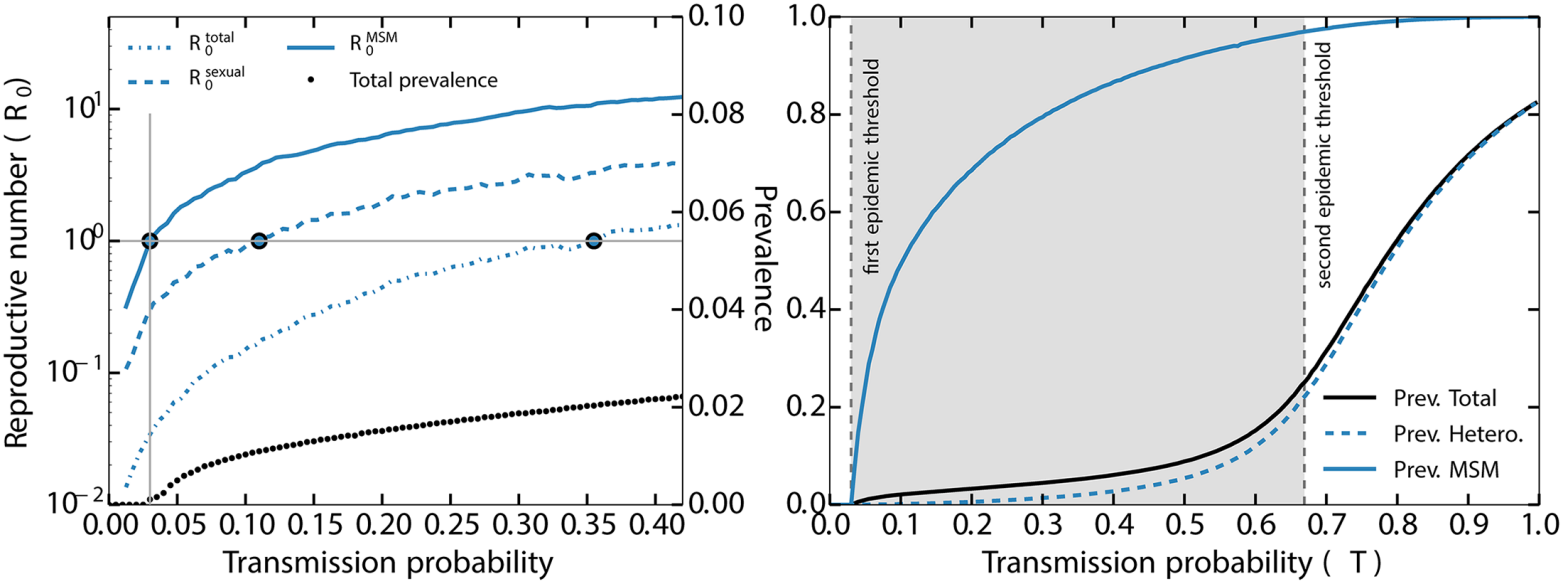
*left panel:* Considering a scenario in which women are 10 times less likely to transmit ZIKV than men (*ε* = 10), the dotted, dashed and solid lines show three estimates of the basic reproductive number *R*_0_ associated with sexual transmissions: (i) $R_{0}^{\text{total}}$ is the average number of secondary infections due to all infected individuals, including by the vector, (ii) $R_{0}^{\text{sexual}}$ is the average number of secondary infections resulting from individuals infected sexually, and (iii) $R_{0}^{\text{MSM}}$ is the average number of secondary infections resulting from individuals in the MSM community who were infected sexually. These estimates are computed by counting the number of sexual infections caused by the individuals infected by the vector and/or their direct neighbors (see Methods). These various *R*_0_ estimates indicate that the transmission probability is at the epidemic threshold when they are equal to one (large dots). The community-specific $R_{0}^{\text{MSM}}$ is the only observable that adequately measures when ZIKV could invade the MSM community; the other metrics falsely imply that sustained sexual transmission is unlikely; critically, $R_{0}^{\text{total}}$ leads to an overestimation of the epidemic threshold, *T*_*c*_, by an order of magnitude (i.e., 0.35 versus 0.03).*right panel:* Prevalence of ZIKV in the whole population and in two sub-populations (MSM and all heterosexuals) as a function of *T* using *ε* = 2 and only considering individuals with more than one sexual partner (as a rough approximation of the sexually active population). The vertical, dashed-gray lines show the threshold values for a self-sustained epidemic *within* the MSM community (with subcritical spillovers in the rest of the population, shaded area), and within the entire population (i.e., supercritical outbreaks exist both within and *outside* of the MSM community), see Eqs (1) and (2). Note that the prevalence outside of the MSM community start rising at the threshold for endemic transmission in the MSM population because of sub-critical spillovers. Note also that we used *ε* = 2 instead of *ε* = 10 for pedagogical reasons since it facilitated the presentation of the results and is clearly conservative with respect to our conclusions.

For a sexually transmitted infection on a contact network, the outcome of having to condition on individuals being infected via a sexual contact is that the critical network metric is not the expected number of transmitting contacts, 〈*k*〉, but is instead the expected *excess* number of transmitting contacts: the number of links other than the one through which they were infected, 〈*k*_e_〉 [42]. In contrast, for individuals who are infected by a mosquito we must use the expected number of transmitting contacts, because the infection was transmitted to them by the vector and not by one of their sexual/social contacts (see Fig 1). This matters because 〈*k*_e_〉 is larger than 〈*k*〉 whenever the variance of the number of transmitting contacts, Var[*k*], exceeds 〈*k*〉. Only for unrealistic contact distribution, such as the Poisson distribution considered by well-mixed compartmental models of ZIKV or a Dirac delta distribution, will 〈*k*_e_〉 be equal to or lower than 〈*k*〉 (respectively). Thus, in all realistic cases, individuals infected sexually are expected to have a greater number of contacts than individuals infected by the vector. And because sexual contact networks are known to be heterogeneous [36, 38, 39], we expect the difference between the two to be quite significant. An accurate assessment of ZIKV’s potential as a self-sustaining STI can therefore only be obtained by explicitly discarding any vector-caused infections in the measurement of $R_{0}^{\text{sexual}}$ (see Fig 1 and the left panel of Fig 2).

### The most at-risk individuals

Because of the asymmetry in infectious period between males (>180 days [8]) and females (<20 days [7]), coupled with evidence for increased infectivity of males [9], it is likely easier for ZIKV to invade the MSM community. Consider two infectious males, one in the MSM community and one not in the MSM community. ZIKV only needs to be transmitted to at least one other individual on average to permit an epidemic in the MSM community, whereas in the non-MSM community, ZIKV needs to infect enough females such that they will together infect at least one other male. In other words, using the terminology described in Methods, we expect ZIKV to invade the MSM community when

$$R_{0}^{\text{MSM}}\;≃\;\underset{men\to \text{men}}{\underset{︸}{T{\langle k_{\text{e}}\rangle }_{\text{MSM}}}}>1\Rightarrow T>\frac{1}{{\langle k_{\text{e}}\rangle }_{\text{MSM}}},$$ (1)

whereas we expect the epidemic to reach the heterosexual population when

$$\begin{array}{c} R_{0}^{\text{sexual}}≃\underset{\text{men}\;\to \;\text{women}}{\underset{︸}{T{\left\langle k_{e}\right\rangle }_{\text{MH}}}}\cdot \underset{\text{women}\;\to \;\text{men}}{\underset{︸}{T{\left\langle k_{e}\right\rangle }_{\text{WH}}/\varepsilon }}>1\Rightarrow T>\sqrt{\frac{\varepsilon }{{\left\langle k_{e}\right\rangle }_{\text{MH}}{\left\langle k_{e}\right\rangle }_{\text{WH}}}}\;, \end{array}$$ (2)

where 〈*k*_e_〉_MSM_, 〈*k*_e_〉_MH_, and 〈*k*_e_〉_WH_ are the average excess number of *potential* disease-transmitting contacts of the MSM community and of the heterosexual men and women, respectively (note that multiplying these quantities by *T* or *T*/*ε* yields the average excess number of *transmitting* contacts). Hence, even if all sub-groups had the same average excess degree (which is generally not the case), we expect two different epidemic thresholds [32]: one for a large-scale epidemic in the MSM community (from which small *subcritical* outbreaks will spillover in the rest of the population); and a higher transmission threshold for an epidemic in the remaining population (see Fig 2). Note also that the stronger the asymmetry in infectious period, the more different these thresholds become, thus departing from the traditional phenomenology of core groups [43]. Indeed, in the case of ZIKV, belonging to the core group does not necessarily imply an above-average sexual activity, but merely a specific sex and sexual orientation (MSM).

To combine the effects of the multiple transmission pathways and asymmetric transmission, we simulated ZIKV transmission on a sexual contact network parameterized with empirical data on contact distributions, population demography, sexual orientation, and disease-specific transmission parameters (see Methods and Fig 2). The left panel of Fig 2 corroborates our previous discussion about the proper way to assess whether a macroscopic epidemic is likely to occur: to consider only sexual transmissions within the MSM community because of ZIKV’s multiple transmission pathways and extreme sex-based asymmetry in the probability of sexual transmission. Furthermore, the results shown in Fig 2 suggest that the threshold for large-scale, sustained sexual transmission could easilybe underestimated by a factor of ten or higher using traditional models. Critically, because the number of sexual contacts within the MSM community is expected to be both higher on average and more heterogeneous than in the heterosexual population [38, 39], we expect a significant gap between the two epidemic thresholds as shown in the right panel of Fig 2. This means that given the high level of asymmetry between probabilities of men-to-women and women-to-men transmissions, there is a potential for sustained epidemics within the MSM community but sub-critical in the rest of the population.

### Surveillance blind spot

Coupled with biases in surveillance, an insidious aspect of the double epidemic threshold of Fig 2 is that one of the most likely outcomes—an epidemic mostly contained within the MSM community—is also the hardest outcome to detect. ZIKV infections in adults are largely asymptomatic [20] and, therefore, most testing occurs in the roughly 20% of cases that are symptomatic or in individuals seeking to have children [44]. The vast majority of these individuals will be outside of the MSM community [44]. However, as stated above, due to male-to-male sexual transmission of ZIKV [4], the group most likely to see sustained sexual transmission is the group least likely to be tested. Further, it will lead to biased estimates in the potential for sustained sexual transmission of ZIKV. Finally, the US FDA restricts individuals who self identify as members of the MSM community from donating blood if they have had sexual contact with another man in the past twelve months [45]. Because donor blood is now routinely, and retrospectively, screened for Zika infection [44, 46, 47, 48], the surveillance situation is further biased against the most at-risk group for sustained sexual transmission.

### Empirical estimation of women-to-men probability of transmission

**Fig 3:**
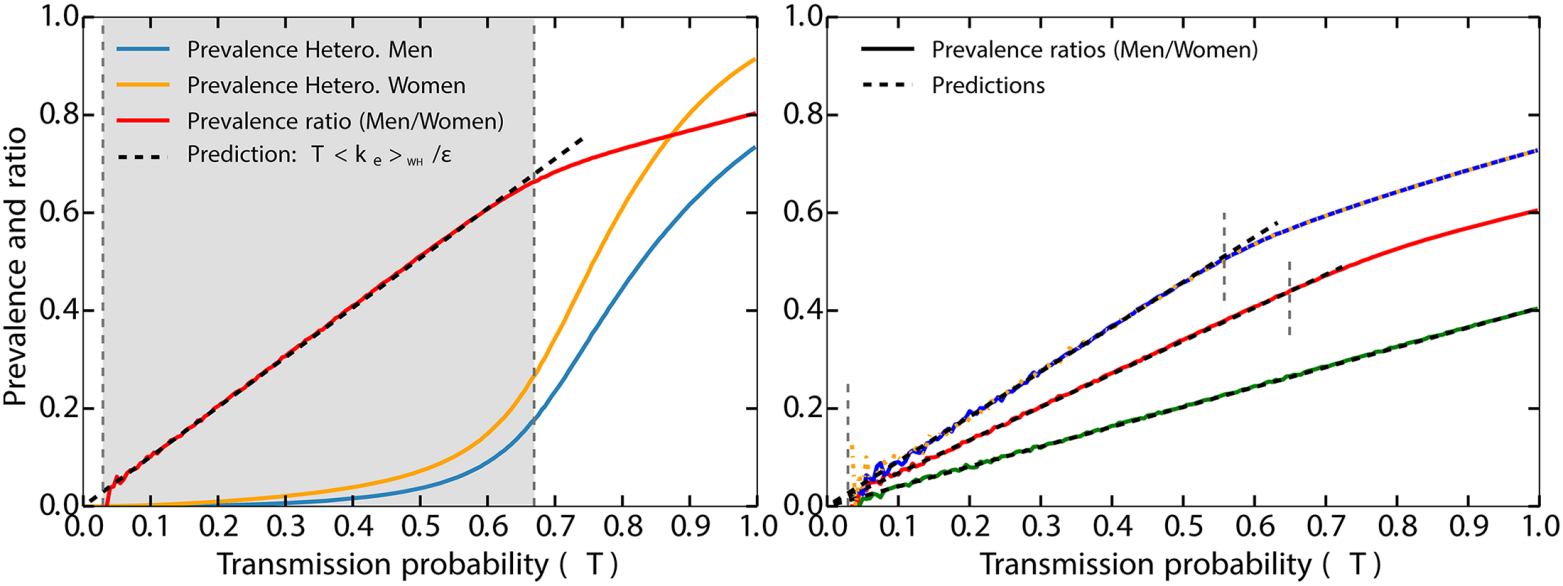
*left panel:* Validation that the ratio of incidence in heterosexual men and women equals *T*〈*k*_e_〉_WH_/*ε*, as predicted in the main text using the same results of numerical simulations as for the right panel of Fig 2. The blue and yellow solid lines show the prevalence of ZIKV within the heterosexual men and women sub-populations, and the red solid line shows their ratio. The vertical gray lines and shaded area are the same as on the right panel of Fig 2. *right panel:* The black dashed lines show the different predictions for the prevalence ratios. The green solid line shows the results of the numerical simulations for the ratio using *ε* = 5 while keeping the same values for 〈*k*_e_〉_WH_ and 〈*k*_e_〉_MH_ as in the left panel (respectively 2.03 and 2.20). The red solid line shows the results for *ε* = 3, an increased value of 〈*k*_e_〉_MH_ (3.51) and an unchanged value of 〈*k*_e_〉_WH_. Notice how the slope increases by a factor 5/3. The blue solid line shows a similar scenario as the red one, but one where 〈*k*_e_〉_WH_ has also been increased (2.75), thus yielding a steeper slope. Finally, the orange dotted line (almost completely under the blue one) shows the same scenario as the blue line, but one in which the average excess degree of bisexual men has been increased (from 5.40 to 7.72 without any effect, as predicted). The vertical dashed gray lines show the different thresholds corresponding to their respective *R*_0_ equals 1 (see Eqs (1) and (2)). Altogether, these results confirm that 〈*k*_e_〉_WH_ and *T*/*ϵ* are the only quantities affecting the risk factor of heterosexual women compared to men.

Between the two epidemic thresholds, there is a sustained epidemic within the MSM community that leads to sub-critical, but recurrent, outbreaks in the heterosexual population. From a network perspective, these outbreaks are bipartite infection trees (from women to men to women to men, etc.) with average branching factors *T*〈*k*_e_〉_MH_ for men and *T*〈*k*_e_〉_WH_/*ε* for women, and always start with a woman who has been infected by a bisexual man himself part of the sustained epidemic among the MSM community. This last observation is important because it implies that a higher incidence in women should be observed in the heterosexual community. Indeed, if heterosexual men could also be at the origin of sub-critical outbreaks, we would expect equal incidence between men and women since both bipartite infection trees have the same combined branching factor, $R_{0}^{\text{sexual}}$. However, for every woman starting an heterosexual outbreak, we have only *T*〈*k*_e_〉_WH_/*ε* heterosexual men continuing that outbreak. This implies that the ratio of prevalence of men to women is expected to be *T*〈*k*_e_〉_WH_/*ε*, which is smaller than one below the second epidemic threshold. This observation is validated in Fig 3 and yields two important implications. First, it tells us that the ratio of incidence between heterosexual men and women is not impacted by the expected excess degree of men 〈*k*_e_〉_MH_. Second, empirical measurements of the prevalence ratio can therefore serve as an estimate of *T*〈*k*_e_〉_WH_/*ε*, which can then be used to estimate the women-to-men probability of transmission, *T*/*ε*, since the average excess degree of heterosexual women, 〈*k*_e_〉_WH_, can be estimated using other traditional means. Evidence supporting these model-based conclusions evidence does exist for ZIKV sexual transmission. Specifically, Coelho et al found a 90% higher incidence of ZIKV infection in women of reproductive age (15 to 65 years old) compared to men, adjusted for gender-related health-seeking behavior and pregnancy status [3].

## Discussion

Sustained sexual transmissions of ZIKV in the MSM community would lead to sporadic sub-critical, but potentially dramatic spillover outbreaks in the heterosexual community, and would increase the probability of endemic establishment of ZIKV. Our result that sexual transmission increases the potential epidemic size and the probability of establishment supports earlier findings from Gao et al. (2016). However, previously published models of sexual transmission do not permit contact heterogeneity nor asymmetry in infectivity. Although we do not study the quantitative effect of heterogeneity, we rather discuss how *R*_0_ should be measured and contextualized when heterogeneity and/or asymmetry exists. Specifically, using a data-driven, but still largely minimal and naive model, we show that the difference in the estimated values of *R*_0_ can lead to drastically different conclusions in terms of the epidemic risk and the probability of disease persistence. Hence, given our evolving understanding of the clinical outcomes for ZIKV infection in adults [49], which, in addition to fever, rash, etc., include autoimmune disorders [50], thrombocytopenia [49], hearing loss [51], and harm to reproductive organs [52] (including risk of fertility issues in males resulting from testicular atrophy [53]), it may be prudent from an individual perspective, as well as a population-level perspective, to increase testing for all adults. Taken together, our results support recent calls to formally consider ZIKV to be a sexually transmitted infection [10, 15, 16, 17, 54].

Public health decision- and policy-makers rely on accurate characterizations of transmission risk to decide on interventions and strategically allocate limited resources. For pathogens, like ZIKV, which are both vectored by an insect and transmitted sexually (with sex-based asymmetry in transmission rate), conventional approaches will underestimate the risk associated with sexual transmission. Underestimating the risk of sexual transmission, both in terms of the average transmission risk and variation in transmission risk, will lead both to biased intervention efforts and to an underestimate of the potential for disease persistence. Additionally, given the differential rates of ZIKV testing in MSM versus non-MSM communities and the known heterogeneity between their contact distributions, the data on sexual transmission are almost certainly biased. We also do not have sufficient experimental or epidemiological evidence for the per-contact probability of transmission in the MSM community. Here we advocate for the decision-making and disease modeling communities to better embrace contact network methods when characterizing transmission risk and for increased ZIKV testing in both MSM and non-MSM communities. The resulting estimates of transmission risk and key epidemiological measures, e.g. *R*_0_ and the likelihood of disease persistence, will be more accurate.
